# Supplementary material for: Are carbon emissions trading and green financial instruments synergistic? -Comprehensive quantitative research based on content analysis
Source: PLoS One. 2024 Mar 7;19(3):e0298601. doi: 10.1371/journal.pone.0298601 (PMC10919601; doi:10.1371/journal.pone.0298601)
Supplement: S1 Data — (ZIP) [file pone.0298601.s001.zip › Data/地级市Z_F工作报告.zip!/║╙▒▒/└╚╖╗╩╨/└┤╫╘╣1⁄2╓┌║┼í╛┬φ┐╦╩2╛▌═°í┐_2006.docx]

各位代表：

现在，我代表廊坊市人民政府向大会做工作报告，请各位代表连同“十一五”规划纲要(草案)一并审议，请市政协各位委员及其他列席人员提出意见。

一、“十五”期间经济社会发展回顾

“十五”时期，是我们面对诸多挑战、经受严峻考验并取得新胜利、跃上新台阶的重要时期。五年来，全市人民在中共廊坊市委的正确领导下，以科学发展观为指导，团结一致，奋发图强，圆满完成国民经济和社会发展各项目标任务，在全面建设小康社会、实现现代化进程中迈出了坚实步伐。

——过去的五年，是廊坊经济发展实现新跨越的五年。

坚持以加快发展为主题，不断增强城市综合实力，经济总量迅速壮大。预计地区生产总值由2000年的368.8亿元增加到2005年的632.9亿元(同口径为680亿元)，年均增长12.1%；2005年全部财政收入剔除基金部分为53.7亿元(同口径为58.5亿元，是2000年的3.1倍)，其中地方一般预算收入24.3亿元，是2000年的2.1倍；全社会固定资产投资五年累计完成1178亿元，年均增长17.5%，是“九五”期间的两倍。

——过去的五年，是廊坊发展环境显著提升的五年。

坚持打基础、造环境，持续实施软硬环境建设工程。累计投资180多亿元，用于交通、供电、通信、环保、水利等基础设施建设。中心城市承载力显著提升，一大批精品工程相继完工。标准化、公共财政、市场监管服务等软环境建设十项工程硕果累累。在全国中等城市中率先通过“ISO14001环境管理体系认证”，成为“ISO14000国家示范区”，获得“中国优秀旅游城市”、“全国文明城市创建工作先进市”、“全国科技进步先进市”、“中国人居环境范例奖”等一批国家级荣誉。

——过去的五年，是廊坊改革开放取得新成果的五年。

坚持把改革力度、发展速度和社会可承受程度统一起来，积极平稳地推进改革攻坚。国有企业、公用事业、农村税费、政府机构、财政体制等各项改革向纵深发展。一批成长性好的民营企业迅速壮大，民营经济增加值占地区生产总值比重达到60%以上。开放型经济快速发展，累计引进内资300亿元，年均增长19.8%，实际利用外资9.5亿美元。环渤海合作机制会议、东北亚暨环渤海国际合作论坛、国际金融论坛年会等具有国际影响的会议定址廊坊。

——过去的五年，是廊坊人民生活水平明显提高的五年。

坚持“以人为本”，注重民生。五年累计新增就业岗位12.5万个，下岗失业人员实现再就业2.79万人，城镇登记失业率保持在2%左右。加快构建大社保体系，养老、失业、医疗、生育、工伤五大社保基金积累达9亿元；住房公积金制度实现全覆盖，累计归集资金12.8亿元。弱势群体救助工作得到加强，困难群众的基本生活有了保障。城镇居民人均可支配收入、农民人均纯收入分别达到10152元和4621元，比“九五”末分别增长49.4%和29.8%。城乡居民人均居住面积分别增加5.3和4.5平方米。

——过去的五年，是廊坊经济社会协调发展的五年。

坚持经济社会统筹，加快各项社会事业发展。人才工作得到加强，科技对经济的贡献率进一步提高，市生产力促进中心成为全省首家国家级示范中心。基础教育水平不断提高，职业教育发展加快，廊坊职业技术学院建成招生。各类文化设施建设投入力度加大，群众文化活动、基层文化建设、专业艺术工作水平提高，一批文艺作品获得国家级奖项。深入实施食品药品放心工程，人民群众饮食用药安全得到保障。全面推进公共卫生体系建设，重大传染病预防控制工作成效明显，抗击“非典”取得重大胜利，防控禽流感工作取得阶段性成果。消除了六个重大火灾隐患，安全生产工作成绩显著。社会稳定工作得到加强。

此外，统计、审计、公安、工商、税务、物价、民政、广播电视、新闻出版、地方志、人防、人口、信访、民族、宗教等工作迈出较大步伐，外事侨务、档案、气象、防震减灾、国防动员等各项工作均取得新成绩。

各位代表，刚刚过去的2005年，是廊坊发展史上不平凡的一年。全市上下把“壮县、强市、富民”作为基础性工作和现阶段主要任务，突出“全党抓经济，重点抓工业，集中精力上项目”，各项工作捷报频传，为“十一五”发展奠定了坚实基础。

1.主要经济指标持续增长。全市地区生产总值增长13.1%；全部财政收入增长27.1%，其中地方一般预算收入增长20%；全社会固定资产投资增长28%，其中城镇固定资产投资增长33.7%；规模以上工业增加值增长24.2%；城镇居民人均可支配收入、农民人均纯收入分别增长10.1%和6.5%。

2.大项目攻坚成果丰硕。全年共实施建设千万元以上项目827项，实际完成投资221亿元，争列省重点项目居全省第一。特别是总投资100亿元的华为北方生产基地、总投资30亿元的中兴北方生产基地、总投资1.2亿美元的利比玻璃、总投资5亿美元的成功(中国)大广场等一批立市立县项目落户廊坊，推动我市项目建设迈上了新台阶。

3.园区经济迅速成长。四个省级开发区实际利用外资占全市的92.7%。廊坊开发区设立出口加工区，成为我市对外开放的第一个国家级品牌。固安、永清等一批新工业园区围绕市场运作、高层次规划和招商机制创新，大胆探索并取得成功，吸引了一批项目落户，成为我市园区经济的新亮点。

4.金融支撑作用增强。金融机构本外币存款余额701.3亿元，比年初增加100.7亿元，同比多增30.6亿元；各项贷款余额391.6亿元，比年初增加57.4亿元，同比多增42.9亿元。建立京津廊票据自动清分系统，实现了京津廊同城结算，在全国首开了支票跨区域流通的先河。成功召开第二届经济金融合作恳谈会，赢得了域外金融机构的资金支持。包括英、德、日、韩等国在内的66家域外金融机构，对我市112家中小企业累放贷款105亿元。争取国家开发银行贷款29亿元，有力地支持了城市基础设施建设和县域经济发展。市农村信用社在全省率先直联大额支付系统，在改革中争取到中央银行专项票据16.5亿元。

5.高科技产业规模壮大。高新技术技工贸总收入达到182亿元，增长17.4%。以华为北方基地、世维光通讯、久智光电子等为代表的电子信息产业群，以量子高科、神威药业、杰希药业等为代表的生物医药产业群，以国瑞电子、鑫谷光电、晶日金刚石等为代表的新材料产业群正在形成。

6.主导产业发展迅速。中国电子百强前十名的华为、中兴落户廊坊，霸州胜芳被命名为中国金属玻璃家具产业基地，汽车零部件产业引进了世原精工、正兴车轮、柳伯安利活塞环等一批知名企业。会展旅游业，全年旅游收入和引进各类展会同比均翻了一番。举办了中国渔具、消防技术设备、石油天然气管道技术装备等一批顶级行业展会，以及20国财长会议(G·20)、京津冀—港澳台(3+3)旅游合作大会等一批国际性重要会议。

7.城建“十大工程”谋划启动。“十大工程”是廊坊建设“百年城市”的基础性工程，是实现市区“一年一变样、三年大变样”的总体谋划，涉及便民利民、城市环境、城市功能、城市经济等四个方面、10大项、54个子项，预计总投资92亿元。目前，龙河工业园区、综合市场改造、经济适用住房等建设工程都已开工，市区水系开发完成测量规划，其它工程都有了实质性进展。

8.“三农”工作进一步加强。落实粮食直补资金4066万元，农业税税率在2004年下调3个百分点基础上再降两个点。全市农业产业化经营率达到57%，汇福粮油、明慧养猪等4家企业跻身国家级龙头行列。全市完成造林33.6万亩。建成文明生态村500个。

9.改革开放成效明显。顺利完成12户国企改制，启动了县级政府机构改革、县乡财政体制改革、农村教育体制改革。华日家具和新奥安瑞科公司在香港联交所挂牌，霸州太阳能采暖公司成为全省首家在美国上市的民营科技企业。省“5·18”经贸洽谈会提升为东北亚暨环渤海国际商务节，中国农交会在市场化办会上迈出新步伐。全年引进内资88.1亿元，增长37.6%；实际利用外资2.64亿美元，增长18.8%。

10.社会进步进程加快。与京津科技合作不断深化，民营科技工作获九项国家级荣誉。提前两年在全省率先普及学前三年教育，北三县(市)和廊坊城区基本普及高中阶段教育，全市高中阶段适龄人口毛入学率提高9.5个百分点。市博物馆、体育健身中心等大型文化体育设施投入使用，一批社区健身工程建成。市疾病预防控制中心和紧急救援中心、8个县(市)疾病预防控制中心建设工程基本完工。“十件实事”和“民心工程”进一步落实，社会保障体系不断健全，全市养老、失业、医疗、工伤保险新增扩面7.7万人；帮助57个无井村近8万农民解决了吃水难；落实农村义务教育“两免一补”政策，解决了50067名贫困家庭学生“上学难”问题；建立健全了流动人口和房屋租赁管理机制；在华北地区率先达到城市交通一等管理水平。“六城联创”成绩显著，成功获得“国家环保模范城”和“国家园林城”荣誉称号，“全国绿化模范城”通过验收。

各位代表，回顾“十五”，业绩辉煌，令人振奋。这些成绩的取得，是省委、省政府和市委坚强领导的结果，是全市上下齐心协力抓发展的结晶，凝结着全市人民的心血和汗水。在此，我代表廊坊市人民政府，向人大代表、政协委员、各民主党派、工商联和社会各界人士以及全市人民，向驻廊中省直单位、人民解放军、武警官兵和政法干警，向关心廊坊、支持廊坊的中外客商与朋友们，表示衷心的感谢！

“十五”时期的工作实践，深化了我们对经济社会发展规律的认识，丰富了我们推进改革开放和现代化建设的经验：一是坚持加快发展。始终围绕抓住机遇、加快发展这一主题，“突出快、追求好”，落实科学发展观，努力实现高效发展、和谐发展、永续发展。二是坚持工业强市。坚持把“工业强市”作为第一方略，大上工业项目，增加工业投入，提高工业化水平，用工业的快速发展推动整体实力的明显增强。三是坚持项目攻坚。牢牢扭住项目建设这个关键，坚持“经济工作项目化、项目工作目标化”，不断加大项目建设和开发开放力度。四是坚持高效服务。以“人居环境甲天下，创业环境国际化”为目标，全力打造廊坊竞争优势，争取服务意识最强、工作效率最高、发展环境最优。五是坚持以人为本。以实现人的全面发展为目标，不断提高人民生活质量，让全体人民共享发展成果，实现经济社会全面协调。

各位代表，在肯定成绩的同时，我们也清醒地认识到当前存在的困难和问题，比如，综合经济实力仍处于全省中游水平，工业支撑作用有待增强；支柱产业尚未真正形成，龙头企业和立市立县大项目少；基础设施和社会事业滞后，“和谐廊坊”建设有待深化；经济发展越来越受到资源和环境的制约，等等。我们必须高度重视这些问题，有针对性地采取措施，认真加以解决。

二、“十一五”的思路目标和主要任务

“十一五”时期，是廊坊经济社会发展承前启后的关键阶段，也是打基础、快发展的重要时期。根据市委三届九次全会精神，在充分调研和广泛征求意见的基础上，市政府编制了《廊坊市国民经济和社会发展第十一个五年规划纲要(草案)》。《纲要(草案)》是全市人民集体智慧的结晶，必将成为指导“十一五”廊坊经济社会发展的蓝图和纲领。

“十一五”经济社会发展的总体要求是：以邓小平理论和“三个代表”重要思想为指导，全面贯彻党的十六届五中全会和省委六届八次全会精神，高扬“树正气、讲团结、求发展”的主旋律，坚持以经济建设为中心，用科学发展观统领经济社会发展全局，按照省委、省政府提出的实现更快更好发展的要求，实施“开放带动、工业强市、科教兴市和可持续发展”战略，把“全党抓经济，重点抓工业，集中精力上项目”进一步抓实，千方百计增加投入，着力培育主导产业，加快工业化、城镇化、农业产业化进程，优化经济结构，壮大综合实力，加快推进“壮县、强市、富民”，全力打造实力廊坊、效率廊坊、和谐廊坊，为“进位·追赶”、率先基本实现现代化奠定坚实基础。

“十一五”经济社会发展的奋斗目标是：全市地区生产总值力争五年翻一番，年均增长14%以上，人均达到4000美元；财政收入达到150亿元，年均增长20%以上，四年翻一番；固定资产投资年均增长20%左右，五年累计投资达到2800亿元。财政收入占地区生产总值比重达到10%以上，单位地区生产总值能耗比“十五”末降低20%左右。全市实际利用外资累计达到20亿美元，年均递增15%以上。城镇居民可支配收入年均增长8%以上，农民人均纯收入年均增长6%以上。

(一)大力培育主导产业。按照“二三一”产业发展格局，着力扶持六大主导产业、八大基地、三十个龙头企业，投资建设500个投资亿元以上大项目，打造一批企业“航母”，形成优势突出、特色鲜明、富有竞争力的产业体系。

(二)调整区域空间布局。着眼于京津冀协调发展和全面接轨京津，加快构筑我市“两带、一轴、一中心”的空间格局。即，沿规划建设的密涿高速公路，开发建设环北京产业带；沿廊泊公路，开发建设环天津产业带；沿规划建设的廊沧高速公路，开发建设中部特色产业轴；以城市功能开发为核心，建设廊坊中心城市，把廊坊建成京津冀、环渤海地区迅速崛起的代表性城市，力争人均地区生产总值排位进入全国地级市百强。

(三)统筹城乡发展。坚持把城市基础设施向农村延伸，城市公共服务向农村覆盖，城市现代文明向农村辐射，逐步建立良性互动、协调发展的新型城乡关系。中心城市“小三点组团(主市区+开发区+万庄新城区)”加快发展，努力推动“大四点组团(小三点+永清+固安+香河)”形成，构筑以廊坊市区为中心，以三河、霸州为两翼，以六个城镇群带为骨干的城镇体系格局。建立健全财政支农资金稳定增长机制，加大对农村基础设施和公用设施的投入，落实各项惠农政策，推进县乡机构、农村义务教育等各项改革，力争40%以上的村建成文明生态村。

(四)扩大改革开放。坚持把改革开放作为经济社会发展的强大动力，形成有利于转变经济增长方式、促进全面协调可持续发展的体制机制。深化所有制和企业改革，推进财政、投融资和行政体制改革，促进民营企业上规模、上水平。加快推进与京津的资源融通、交通互连、市场互通、信息共享、产业互补，构筑全方位、宽领域、纵深化的对外开放格局。

(五)优化发展环境。坚持把环境建设作为第一竞争点，深入实施环境建设重点工程。硬环境建设重点是：交通网络建设，总投资100亿元以上，平均每年投资20亿元以上，重点建设4条高速、7条干线公路，实现县县通高速、县城通一级路、乡镇通二级路、村村通油路。能源建设，到2010年，全市将运行500千伏变电站2座、220千伏变电站17座，形成可靠性高并适度超前的电网。水利设施建设，加快推进南水北调配套工程以及防洪、蓄洪、除涝工程建设，重点完成廊坊干渠(涿州—廊坊广阳水库)、天津干渠(徐水—天津)、广阳水库建设工程。软环境建设重点是：以建设“效率廊坊”为目标，积极推进政务改革，塑造亲商、近商、安商、富商的社会环境。加快实施标准化体系、信用体系、中介服务体系“三大体系”建设，着力打造健康规范的市场环境。加强人才引进与培养，建设“人才高地”。进一步深化金融网络、金融产品和金融服务“三大创新”，推进银企良性互动。

(六)加快“和谐廊坊”建设。建设创新型城市，以产业为基础，以企业为主体，以京津为依托，走廊坊特色的自主创新道路。加强教育、文化、体育、卫生等基础设施建设，提高全民受教育程度，努力让城乡居民享受到优质、高效、低廉的医疗卫生服务。统筹解决人口素质和结构方面的突出问题。进一步扩大基本养老保险、医疗保险、失业保险、生育保险、工伤保险、城乡最低生活保障覆盖面。完善食品安全监督、安全生产监管、社会矛盾排查调处和社会治安防控体系，不断提高政府保障公共安全和处置突发公共事件的能力。建设资源节约型、环境友好型社会，提高水的开发和利用率，推广集约和清洁生产，发展循环经济，积极创建生态市。推进社会主义精神文明和民主法制建设，进一步落实《公民思想道德建设实施纲要》，大力开展群众性文明社区、文明村镇、文明单位等创建活动，深入开展法制和科普宣传教育，巩固和加强基层民主建设。

三、2006年的主要工作

2006年是实施“十一五”规划的开局之年，是加快推进“壮县、强市、富民”的关键一年，是各项工作谋求新突破、再上新台阶、实现大发展的重要一年。2006年全市经济社会发展的主要预期目标是：地区生产总值增长14%左右；全部财政收入增长20%以上，其中地方一般预算收入增长16%；全社会固定资产投资增长20%以上；实际利用外资3亿美元；城镇居民人均可支配收入增长10%以上；农民人均纯收入增长6%以上；城镇登记失业率控制在3.5%以内。

做好今年的工作，关键是围绕打造“实力廊坊、效率廊坊、和谐廊坊”，完成三大任务，做好十方面工作，确保“十一五”开好局、起好步。

完成三大任务：一是抓工业、上项目、招商引资，力促经济发展更快更好；二是创条件、造环境、提高效率，增强城市软实力；三是抓协调、补弱项、富民优先，建设和谐新廊坊。

做好十方面工作：

1.落实“两抓一上”，打造工业强市。

廊坊发展的主导在工业，出路在工业。落实“全党抓经济，重点抓工业，集中精力上项目”，重点是抓好六大产业(基地)、十大园区和三十个超5亿元大项目。着力推进以高新技术产业为主的制造业基地建设，培育知名品牌，打造知名企业。

培育六大产业，即电子信息、汽车摩托车零部件、金属制品、木材加工及家具制造、食品加工和会展旅游产业，相应建设六大基地。

提升十大园区，即廊坊、燕郊、香河、霸州四个省级开发区，固安、永清、龙河三个工业区，大城摩配、文安胶合板两个科技园区和大厂民族工业园区。

谋划实施三十个超5亿元大项目，即开工建设固安移动平板显示基地、三河电厂二期、文安环保产业基地、霸州德利国际新能源示范基地等十个重点项目，新谋划二十个超5亿元大项目。

2.突出招商实效，推进“招商引资突破年”。

做大总量、调优增量，重点在工业，出路在开放。着眼于以大开发促大开放，以大招商促大突破，营造全员招商氛围，全面提升招商实效，确定2006年为“招商引资突破年”。

造浓招商氛围。树立招商引资是“第一政绩”、“第一能力”的观念，使之成为每个县(市、区)、每个部门的硬任务，言必说招商，行必抓招商，形成大密度招商、大力度招商、全员招商的浓厚氛围。

突出定向招商。组织开展“十百万千”活动，即举办十项重大招商活动、组织百团出访招商、拜访和接待万名客商、签约千个项目。各县(市、区)、各园区根据自身实际，瞄准重点领域、主攻区域，形成连绵不断地强势冲击。年内组织珠三角、长三角、港、台、欧、美、韩等专项招商活动。

创新招商方式。强化市场化招商，按照国际通行做法，委托专业机构招商，提高项目成功率；强化产业招商，重点组织好电子信息、汽车零部件等产业招商活动；强化以商引商，用优越的投资环境增强现有客商对廊坊的信心；强化企业招商，动员组织各类企业外出招商，对其新增招商项目和销售收入给予政策优惠。

强化责任机制。出台《廊坊市关于进一步加强招商引资工作的意见》，对各级各部门实施项目招商目标责任制，把招商责任具体到每个单位、传递到每个人，明确招商引资的目标和任务，按季度通报招商引资完成情况，全年发布排行榜，对做出突出贡献的集体和个人实施重奖。

3.依托主导产业和工业园区，集中精力上项目。

围绕推动项目总量和质量双提升，突出“四抓”，在建项目抓重点，谋划项目抓产业，保障项目抓机制，承载项目抓园区。

在建项目抓重点。加快128个投资超亿元重点项目建设进度，确保早开工、早投产、早见效。对华为北方生产基地、中兴北方生产基地、固安移动平板显示基地、成功(中国)大广场等一批投资10亿元以上重大项目，实行市级领导负责制，逢山开路，遇水架桥，全力确保。

谋划项目抓产业。围绕主导产业，盯住国内外业内知名企业，谋划实施一批超5亿元大项目。围绕华为北方生产基地等立市立县项目，狠抓配套引进，培育项目群，拉长产业链。积极争列一批省重点项目。不断充实各级项目库，确保项目接连不断，后劲充足。

保障项目抓机制。继续组织项目拉练，发挥北京、天津、石家庄三个项目办事处的作用，提高投资项目服务中心的运行效率，建立重点项目目标管理责任制。加大项目前期工作投入，市本级新增1000万元。对列入省重点计划的项目，实行“五定”责任制，即定单位、定投资、定工期、定人员、定奖惩，层层抓落实、步步见脚印。

承载项目抓园区。提升对园区工作的认识，不断加大园区开发力度，下大力解决好观念不新、机制陈旧、规划水平低等突出问题。重点是“四个提升”：提升园区开发水平。廊坊开发区加快扩区步伐，争取成为国家级开发区；廊坊出口加工区加快规划建设，年底通过国家验收，实现封关运行；燕郊集中精力开发韩国工业园，年内打好基础、形成规模；固安、永清乘势而上，努力实现项目建设新突破；龙河工业园借鉴先进园区的新理念，高起点起步、高水平建设；香河按一流水准建设新园区，启动基础设施，争取引入项目。提升园区规划水平。各园区根据自己的实际情况，聘请国内外高水平专业机构对各园区规划进行修订完善。提升园区服务水平。进一步完善功能配套，在招商前期、创业注册、吸引措施、人才技术支持、投资融资等相关领域更新政策、创新服务。提升园区基础设施水平。市向县一级转贷5亿元国家开发银行贷款，集中用于园区基础设施建设。各县(市、区)广辟资金渠道，鼓励有条件的企业参与园区建设管理。

4.加快主导产业发展，增强产业支撑力。

围绕六大产业，在理顺体制、搞好规划、抓好配套上下功夫。成立产业发展局，谋划产业发展，加强协调指导。分别建立产业推进领导小组，市级领导负责，做到一个产业一套人马、一个规划，一抓到底。出台廊坊市产业发展规划，制定六大产业具体规划，采取有效措施，延长产业链条，壮大产业集群。

建立完善自主创新体系，推动主导产业升级。重点抓好“三个强化”：强化创新主体，推动企业技术开发中心建设，力争1—2家进入省级以上重点实验室管理序列。强化创新体系，依托京津和驻廊高校院所，以清华科技园、中科院产业基地等为载体，加快关键技术的引进吸收和再创新，构建开放式区域创新体系。强化创新平台，以制造业信息化技术推广中心、生产力促进中心等为载体，为中小企业提供共性技术服务；以燕郊和廊坊高新区创业中心等为载体，加快高科技成果转化、孵化；以科技创业投资公司为载体，撬动域外资本向廊坊聚集。力争通过三年努力，建成一批销售收入超百亿的产业集群。

电子信息产业。发展环北京电子信息产业集群，建立完善通讯设备、液晶面板配套件、半导体、计算机及其外设等产业链，发展电子材料、视听整机及通讯设备等五个生产加工基地，推进华为、中兴、豪威、博为等一批龙头项目，抓好项目配套，加快产业集群化进程。全年实现增加值10亿元，增长20%。

汽车摩托车零部件产业。依托廊坊、燕郊、香河开发区和固安工业区、大城摩配科技园区，大力引进一批新项目；加快推进香河展望、正兴车轮、世原精工等一批在建项目。全年实现增加值11.55亿元，增长24%。

金属制品产业。着力提高产业集中度和技术水平，重点建设薄壁钢管、金属包装容器和金属结构三大制造基地。全年实现增加值22亿元，增长22%。

木材加工及家具制造产业。抓好顺德家具、八达家具等项目建设，促进产品结构向高端延伸，提高产品附加值，提升档次，打造名牌。全年实现增加值32.1亿元，增长20%。

食品加工产业。充分发挥好丽友食品、伊利奶业、新瑞番茄等品牌优势和市场引领作用，做大做强汇福粮油、福成五丰、梅花味精等一批龙头企业，开发深加工、精加工项目，带动上下游企业的升级。全年实现增加值48.1亿元，增长21%。

会展旅游产业。抓好国际饭店B座、恒盛阳光、愉景温泉等五星级酒店建设和天都扩建升五星工程，新增一批旅游项目，引进一批国内外知名展会。全年力争实现会展旅游收入37亿元，增长18%；举办大型展会数量在2005年翻番的基础上力争增长50%。

5.打造品牌城市，增强城市软实力。

打造实力廊坊，既要增强城市的综合经济硬实力，也要增强以城市环境、城市文化、城市品牌为代表的软实力。在当今世界的城市竞争中，软实力尤为重要，它反映一个城市的核心竞争力，决定着一个城市长远的发展后劲。着眼于增强对项目资金人才的吸引力，促进主导产业发展，增强城市软实力，当前主要做好五个方面工作，即优化软、硬两个环境，富集金融、人力两种资本，加强城市营销。

硬环境：以城乡基础设施建设为重点，全力抓好五项建设。

市区“十大工程”建设。以打造“百年城市”为理念，以建设精品工程为目标，集全市之力推进“十大工程”建设，进一步提升中心城市功能。按照“三年大变样”总体要求，2006年“十大工程”全面启动，确保一批工程竣工。年内开工建设阳光政务中心、人民公园升级改造、市体育场、路网完善提升等29个子项目。其中10条道路、6个市场等21个子项目竣工，龙河工业园区和万庄新城区建设取得初步成效，人民公园中国农交会前向市民免费开放。

市区水系建设。着眼于营造城市水环境，提高城市品位，让廊坊真正成为“有水”的城市。对龙河、凤河等河渠进行总体规划和综合治理，实现河渠相通、水水相连，营造碧水绿岸、自然怡人的城市休闲空间。今年开始，重点对龙河、大皮营引渠、八干渠进行截污、清淤、扩挖、造景，确保市区水系工程取得实质性进展。

“绿化精品”建设。突出大投入、力求高档次，做到乡村绿化出亮点、城市绿化出精品。乡村绿化重点建设生态防护林和生态景观带，以“三北”、“退耕”、“治沙”等工程林为主体，完成造林30万亩。城市绿化突出抓好新华路、金光道、广阳道等5条精品线，完成西、南外环绿化工程和10个游园、文化艺术中心二期景观工程。市区集中栽种桧柏、雪松、黄杨、法桐、四季海棠等适宜花木，营造“三季有花、四季常青”，独具北方特色的绿化景观。市区绿化覆盖率达到45%，人均公共绿地面积达到9.5平方米。

交通设施建设。完成市区西外环南延、光明西道西延、郊区快速路北段工程，开工建设唐通线香河县城至北京段。开工建设廊涿、大广两条高速，谋划并争取开工建设廊沧高速、102高速、京沪高速连接线、廊涿高速固安和永清连接线5个项目。完成县乡道路改造180公里。

能源建设。启动三河电厂二期工程，开工建设高楼、高头220千伏输变电工程，以及金辛庄、新镇等8个110千伏输变电工程，进一步提高电网供电能力。加快大城煤田勘测开发进度。

软环境：以改善政务环境、提高服务水平、降低商务成本为核心，提升廊坊软实力。

提高政府办事效率。把行政审批中心建成方便市民的政务超市、企业发展的服务中心、投资环境国际化的展示平台。审批项目再精简，服务效率再提高。建立大项目“绿色通道”，由发改委及国土、环保、规划、建设等部门联合办公，及时解决大项目申报建设中的各种问题。成立项目代理服务中心，对投资项目立项、审批、办证、缴费等各类事项，全程代办，直通服务。建立完善行政信息平台，整合相关信息，各类行政决策事项、非涉密文件及时向社会公布，实现资源共享。

强化行政效能监察。成立市效能监察投诉中心、民营企业投诉受理中心。对行政效能投诉，逐件登记查办，限时办复。开展明查暗访，对不作为和乱作为现象据实查处。

清理整治罚没收费。规范罚没收费行为，严格实施“罚缴分离”和“收支两条线”管理，坚持不下达罚款指标和零返还政策，执法经费由财政全额划拨。全年罚没收费总额力争明显下降。努力把廊坊建成京津冀地区收费项目最少、标准最低的城市。

金融资本：以优化金融生态环境、拓宽融资渠道为重点，增强金融对经济的支撑作用。

努力增加信贷投放。搭建银企对接平台，适时召开政、银、企恳谈会，促进银企沟通与协作，吸引域外金融机构来廊开展业务或建设分支机构，拓宽企业融资渠道。搞好农村信用社改革，争取尽早兑付中央银行票据。推行中小企业联保贷款、中小企业最高额抵押贷款、优质客户贷款、助学贷款、下岗失业人员小额担保贷款等，增加资金支持。研究发展民间信用，规范民间融资，探索发展各种融资基金，促进企业直接融资。

改善金融生态环境。制定金融生态环境评价指标体系。开发金融综合业务网络平台，方便银行、企业和居民办理一般结算、代收代付等业务。加快信用体系建设，整合金融、劳动、工商、税务、公安等各方面资源，基本建成个人信用信息基础数据库，完善企业信用信息数据库，研究确定企业信用评价指标体系，加快信用产品的推广应用。

人力资本：以引进、培训为重点，加大开发力度，为经济发展提供充沛、优质的人力资源。

推进人才高地建设。引进人才调结构，培养人才上层次，使用人才上水平，实现“4832”目标。侧重项目、产业、园区和非公企业，每年引进400名博士、硕士、高级职称或高技能人才，组织800名机关事业单位人员参加在职和脱产学历教育，组织3000名公务员参加各种培训，人才柔性流动2000人次。年底，全市人才密度达到12.72%。

加强职业技能培训。以新型农民培训、实用技术培训、各类证书培训和学历培训为主，全年培训农民30万人，确保年底完成百万农民培训工程。建立培训与使用机制，使更多农民通过培训进入非农产业。加强规划统筹、政策统筹、办学统筹，发展职业介绍机构和职业培训组织，打造高素质的职业技工队伍。

城市营销：以城市管理做精、做优、做美为基础，以打造品牌城市为目标，实施城市营销计划。

加强城市管理。坚持做精、做优、做美的理念，重点在城市规划、城市管理和社区建设上下功夫，努力彰显城市特色。推行高标准规划，设计建设一批精品工程，完善城市功能，提升城市品位。加强城市综合执法，完善长效机制，开展系列城市环境整治活动。研究引入高技术手段，探索实施“数字化”城市管理。把社区建设作为城市管理的基础工程，加强机构、人员和资金保障，建设社区行政事务受理中心和服务中心，丰富“十五分钟生活圈”内涵，推进“平安社区”建设，促进由单纯的居住区向社会功能完善的社区转变，由单纯的物业管理向多方位服务转变，逐步实现事事有人管、人人来参与、城市更和谐。

打造城市品牌。导入城市设计理念，深入研究市情，整合各类要素资源，制定品牌战略，搞好前期策划和总体规划，突出重点，分步实施。从经济、文化、教育、卫生、体育、安全、生态等多方面入手，挖掘提升老品牌，培育打造新品牌。在此基础上，进一步培育城市特色，使廊坊成为品牌城市。

开展城市营销。制定整体营销计划，启动系列营销活动。开展会展营销，通过“5·18”国际商务节、中国农交会等重大招商活动，以及重大体育赛事和文化活动扩大影响。加强宣传推介，到招商引资重点区域，利用国内外主流媒体和互联网，开展系列宣传活动，展示城市整体形象。

6.坚持富民优先，激活民营经济。

民营经济是廊坊的富民经济、主流经济。贯彻落实中央《关于鼓励支持和引导个体私营等非公有制经济发展的若干意见》，坚持外抓招商、内抓民营，培育全员招商、全民创业的浓厚氛围，让民众的创造力、民企的扩张力、民资的爆发力最大限度地释放出来。力争民营经济营业收入增长18%以上，上缴税金增长16%以上。重点实施四项工程：

全民创业工程。制定实施“民营经济腾飞计划”，把全民创业作为壮大民营经济、实现强市富民的基础性工程，在全社会宣传创业理念、培育创业文化、树立创业导向，让更多的创业者放心创业、创成大业。对自强自立、拼搏进取实现再就业的下岗失业人员，以及各类经济组织创业业绩突出人员，进行大张旗鼓的表彰，使全民创业蔚然成风。

上档升级工程。按照企业发展规律，选择一批潜力大、成长性好的中小企业，加强引导规范，实施公司化、股份制改造，促其上水平、上档次，进行“二次创业”。围绕主导、特色产业，整合行业生产能力，鼓励强强联手、资产重组、股份合作，组建一批企业集团，做强一批龙头企业，打造规模和集群优势，迅速提高企业层次。

创新服务体系工程。搭建创业服务平台，新建一批小企业孵化、成长基地。搭建人才培训平台，利用现代化远程教育等手段，全年完成“万人培训”目标。搭建融资服务平台，多渠道搞好银企对接，鼓励民间资金和省外资金进入担保领域，形成20亿元的担保能力。

政企交流畅通工程。建立信息通报制度，发挥行业协会作用。通过论坛、沙龙、早餐会等形式，搭建各级政府领导与企业家的对话平台，增进相互沟通，密切政企关系。建立领导干部与企业家联系制度，每名领导干部联系一两家民营企业，公开联络方式，建立绿色通道，形成解决企业难题的快捷方式。畅通投诉渠道，及时受理企业投诉举报，规范有关部门涉企收费和执法行为。

7.大力发展农村经济，建设社会主义新农村。

按照“生产发展、生活宽裕、乡风文明、村容整洁、管理民主”的要求，以创建文明生态村为载体，扎实推进社会主义新农村建设。

创建文明生态村。以壮大集体经济为基础，以发展农村经济和增加农民收入为主线，按照“环城式、组团式、连线式”模式，创新机制，梯次推进。争取年内再建成400个文明生态村。

加大对农村的扶持力度。实现由“少取”到“多予、放活”的转变，加大财政支农力度，安排新农村建设专项资金4000万元，重点抓好农村“路、水、保、养、医、学”六项工程。继续实施村村通油路工程，新修油路328公里，新增通油路村298个，实现村村通油路目标。实施人畜饮水安全工程，自来水普及率达到85%，解决60个村、4.2万人的饮水困难，3—5年全面解决人畜安全饮水问题。实施农村社会保障工程，全面启动农村最低生活保障，出台《廊坊市农村最低生活保障实施细则》，总结试点经验，努力实现全覆盖；完善失地农民社会保险办法，切实解决好失地农民的生活保障问题。实施农村“五保户”集中供养工程，加快农村养老院建设，在全省率先实现半数以上“五保户”集中供养。实施农村医疗惠民工程，抓好城乡医疗救助工作，出台《医疗救助实施意见》，实现操作规程、资金使用的规范化、合理化；完善三河新型农村合作医疗运行机制，全面启动霸州、香河新型农村合作医疗试点；完成20所乡镇卫生院改扩建，积极建设达标村卫生室。实施农村义务教育“控辍保学”工程，开展社会捐资助学和孤残儿童救助活动，认真落实“两免一补”，确保不让一名学生因贫困失学；推进农村中小学危房改造，三河、广阳、香河、霸州、文安今年全部消除现存D级危房。

推进农业产业化经营。培育加工龙头企业，延伸产业链条，提高农民组织化程度和农业综合效益。农业产业化经营率达到58%，提高1个百分点；销售收入500万元以上加工型龙头企业达到150家，争取增加4—5家省级龙头企业；市一级重点发展10个种植业基地、10个养殖业基地，扶持5个农产品加工示范基地，发展40个农民专业合作组织。

发挥城镇带动作用。高标准建设小城镇，充分发挥城镇对农村建设的示范带动作用。创新胜芳管理模式，提高规划、建设、管理水平。全面启动镇、村两级规划，完成牛驼、渠口等20个乡镇的总体规划修编。实施城镇基础设施精品示范工程，加强各县城路网等基础设施建设，每个县(市)建设1—2条精品路。

8.深化各项改革，推动体制机制创新。

深化改革是加快发展的动力，制度创新是增强实力的源泉。借鉴先进地区经验，大胆探索，重点在四个领域实现突破。

打好国企改革攻坚战。按照建立现代企业制度的要求，加快国有企业的股份制改造，鼓励非国有企业、投资者参与国有企业改制。加快流通体制改革，出台《廊坊市加快流通业发展实施意见》。年内完成外贸包装、恒宇工具等8家市属企业，大厂冷冻厂、永清农机修造厂等13家县属企业改制。

理顺国有资产监管体制。明确国有资产监管机构与各单位的监督管理关系，落实国有资产保值增值责任。探索县级国有资产监管运营的有效形式，逐步形成全面覆盖与重点监控并重、统一管理与委托授权并举的国有资产监管新模式。开展国有企事业单位清产核资、产权登记、产权界定、资产评估等工作，加强国有产权转让行为监督，促进国有产权规范有序流转。

积极推进社会事业和公用事业改革。引入市场机制，研究制定鼓励社会办事业的政策，引导社会资金进入医疗、卫生、教育领域，推动条件成熟的医院和非义务教育学校加快改制步伐。按照“分类定位，分开管理，分别改革”的思路，进一步推进城市道路、供水、排水、供热、垃圾处理、污水处理、市容环境卫生和城市绿化等方面的改革进程，三年内改制面达到95%。

鼓励企业上市。按照“上市一批、推荐一批、培育一批、储备一批”的思路，落实责任，加强扶持，鼓励和推进梅花味精、汇福粮油、华洋线缆、明慧养猪等优势企业实施股份制改造，培育上市梯队。2006年，争取1家企业上市融资，1—2家企业进入上市辅导阶段，1家上市公司完成股权分置任务，促成1家已上市企业实现再融资。

9.以人为本，推进“和谐廊坊”建设。

更加注重社会事业发展，更加注重社会公平和安全稳定，更加注重生态环保建设，深入建设“和谐廊坊”。

加快各项社会事业发展。科技工作重点抓“一城、一带”，即全力创建全国科技示范城，加快发展高科技产业带。全市高新技术产业增加值达到53.8亿元，占地区生产总值的比重达到7%。教育工作坚持“义务教育抓均衡、职业教育扩规模、民办教育上水平”。促进义务教育均衡发展，努力缩小城乡之间、区域之间、学校之间的差距；以中等职业教育为重点，扩大职业教育招生规模，增加中等职业教育在高中阶段教育中的招生比重，高中阶段教育毛入学率达到76.6%；大力发展民办教育；全面实施素质教育。加强公共卫生体系建设，进一步提高疾病预防控制、卫生监督执法和突发公共卫生事件应急处置能力；完成市传染病医院、市医院改扩建，推进市妇幼保健中心和总投资11亿元的燕郊东方国际医院工程。抓好文化精品建设，保证图书馆年内投入使用。完善全民健身服务体系，力争在省十二运会实现“保六争五”目标。落实“计生惠民”政策，完善计划生育利益导向和长效工作机制，加强“性别比”问题治理，严肃查处非法胎儿性别鉴定、非法终止妊娠案件。

深入实施“民心工程”。牢固树立发展为了人民、发展依靠人民、发展成果人民共享的观念，着力解决好人民群众最关心、最直接、最现实的利益问题。做好就业再就业工作，建立城乡统筹的劳动力市场，全年新增就业岗位2.8万个；实施农村劳动力转移培训阳光工程，提高农民素质，转移农村剩余劳动力10万人，劳务输出6万人。扩大社会保障覆盖范围，全年新增参保6万人，住房公积金年度归集3.5亿元。加大经济适用房开发力度，全年开竣工面积达到20万平方米，解决中低收入者住房问题。加大市场建设力度，强化市场监管，整顿和规范市场经济秩序，积极推进产品质量电子监管网建设。实施食品药品放心工程，建立完善农产品质量标准、质量安全检测和认证体系。

狠抓安全生产和社会稳定。健全长效监管和应急救援两大体系，建立安全生产投入、教育培训和责任追究三项机制，在县乡村三级健全机构、配足人员，在生产经营单位设置机构和专兼职安全管理人员，确保安全生产。继续推进“十百千”工程，开展“无越级访单位”创建活动，每月进行排查，把矛盾纠纷化解在萌芽、解决在基层。畅通不同利益群体的诉求渠道，建立完善信访听证、预警通报等工作机制，积极预防和妥善处置群体性事件。加强社会治安综合治理，完善打防结合、预防为主、城乡一体的社会治安防控体系。加强突发公共事件应急管理，落实《突发公共事件总预案》，完善预警、分析、救援、决策、指挥系统，提高应对突发公共事件和抵御风险的能力。

加强生态环境保护。认真落实国务院《关于落实科学发展观加强环境保护的决定》，超前谋划，科学规划，启动生态市、联合国臭氧层友好城市创建工作，推动固安、文安、香河、三河等县(市、区)建设生态示范区和环境优美乡镇。深入实施环保“蓝天、碧水、净土、绿色、宁静”五大工程，确保ISO14001环境管理体系的有效运行，巩固和提高“国家环保模范城”和“ISO14000国家示范区”创建成果。市区空气质量二级以上天数330天以上，确保集中饮用水水质达标、污水处理水质达标，市区医废处置中心和各县(市、区)垃圾处理场、污水处理厂建成使用。出台《廊坊市中水设施建设管理规定》。

10.加强政府自身建设，不断提高行政能力。

按照为民、务实、清廉的要求，提高行政能力和管理水平。

提高驾驭市场经济的能力。掌握市场和产业发展规律，注重全局谋划和总体把握，增强和改进对重大事务的综合协调能力。进一步创新工作方法，善于用经济杠杆和法律手段抓发展。

提高抓落实的能力。从作风上促进抓落实。把主要精力引导到干工作、抓落实上来，善于抽出时间深入基层，到一线开展调研，有针对性和创造性地开展工作；深入群众，察民情、听民意、解民忧，为基层和群众解决一批实际困难和问题。在机制上保障抓落实。强化目标责任制，做到一级抓一级、人人有专责、事事有人管；强化督导检查，对重大决策部署和重要事项的落实组织专项督查、专题调度，一抓到底；强化考核奖惩，对重点工作重点考核，对群众关心的重要事项开展社会评议，严格落实奖惩措施。

提高依法行政能力。全面落实《行政许可法》和依法行政《纲要》，努力做到合法行政、程序正当、高效便民、诚实守信、权责统一。加强科学民主决策目标体系、执行责任体系和考核监督体系建设。自觉接受人大法律监督和政协民主监督。以《公务员法》施行为契机，建设高素质的公务员队伍。

提高廉洁行政能力。建立健全教育、制度、监督并重的惩治和预防腐败体系，着力治本，注重预防。继续全面落实廉政准则和党风廉政建设责任制。前移廉政关口，加强廉政教育，筑牢思想防线。完善用权、用人、用地、用钱等方面的制度，强化机制约束。完善农村财务委托代理等监管办法，加强农村基层党风廉政建设。加大对各类违法违纪案件的查处力度。

各位代表，站在新起点，实现新发展，是时代赋予我们的光荣使命和神圣职责。让我们在中共廊坊市委坚强领导下，紧紧团结和依靠全市人民，凝聚各方面的智慧和力量，攻坚克难，开拓进取，万众一心，扎实奋斗，谱写廊坊更加辉煌的新篇章！
